# Supplementary figures and images for: The chromatin insulator CTCF regulates HPV18 transcript splicing and differentiation-dependent late gene expression
Source: PLoS Pathog. 2021 Nov 4;17(11):e1010032. doi: 10.1371/journal.ppat.1010032 (PMC8594839; doi:10.1371/journal.ppat.1010032)

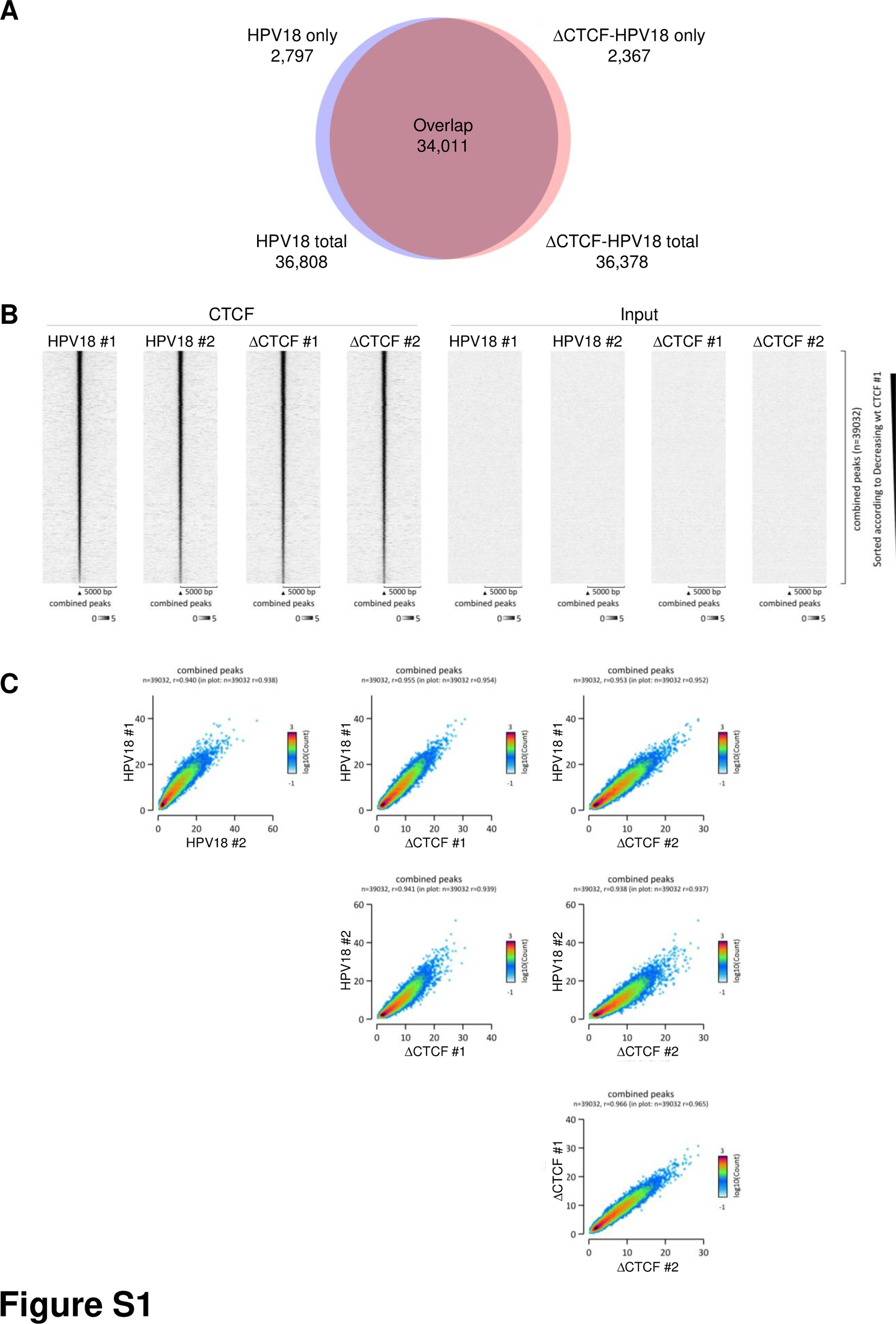

Supplement: S1 Fig — (A) Venn diagram of CTCF peak regions in cells containing either HPV18 or ΔCTCF-HPV18 episomes showing the average total number of peaks present in two independent replicates within each condition as well as the number of overlapping and unique peaks. (B) Heatmap visualization of CTCF ChIP-Seq replicates from two independent HFK donors (#1 and #2) and corresponding input sample centered on the combined peak regions detected in HPV18 and/or ΔCTCF-HPV18 samples. (C) Scatter plots of pairwise sample comparisons show high correlation between replicates as well as between HPV18 and ΔCTCF-HPV18 samples. Pearson’s correlations coefficients (r) are given in the plots and are above 0.93 in any pairwise comparison. (TIF) [file ppat.1010032.s001.tif]

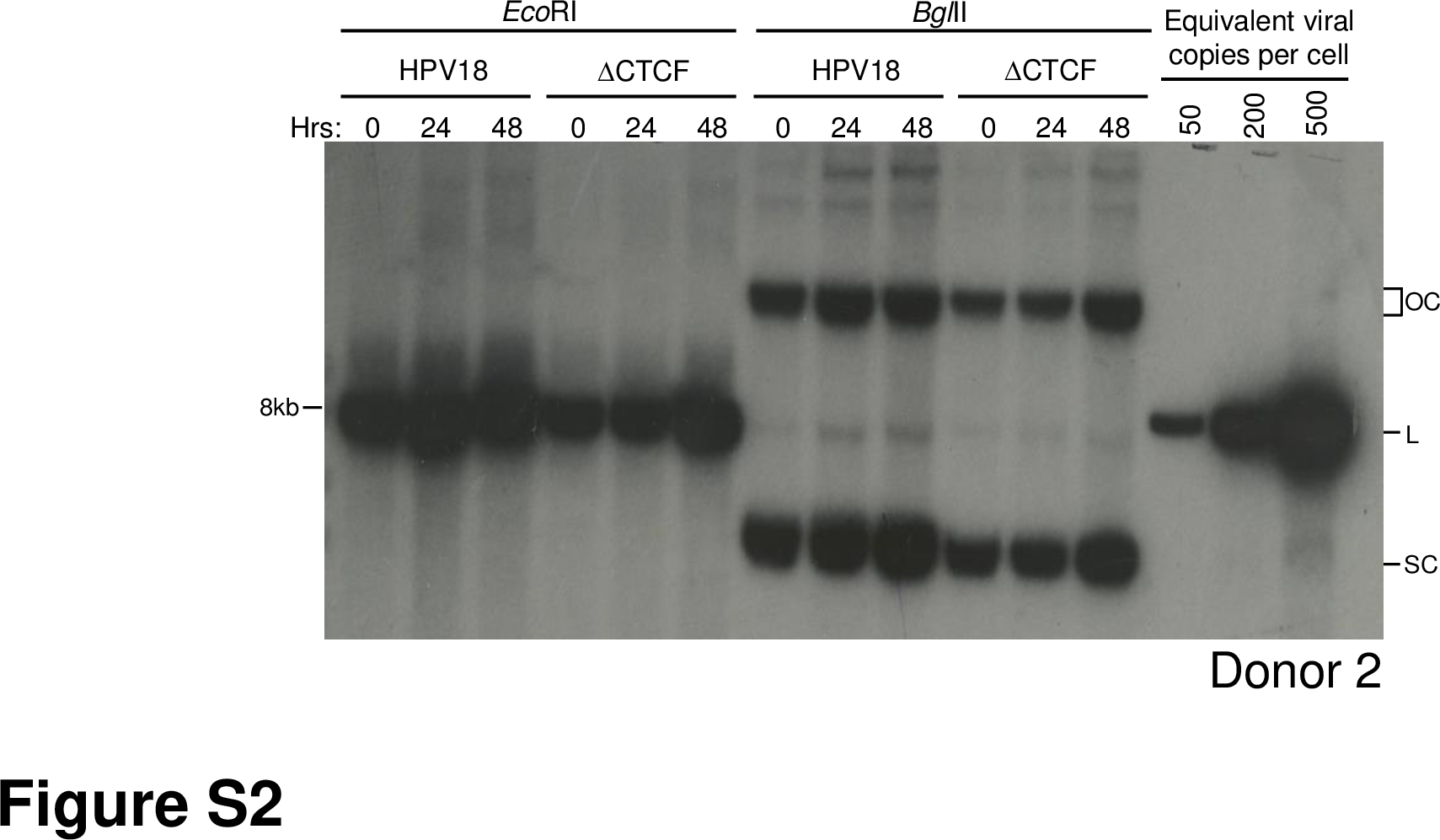

Supplement: S2 Fig — Amplification of HPV18 and ΔCTCF-HPV18 episomes was detected by Southern blotting following digestion with EcoRI to linearise the HPV18 episomes, or BglII which digests cellular DNA only (OC, open circle; L, linear; SC, supercoiled). (TIF) [file ppat.1010032.s002.tif]

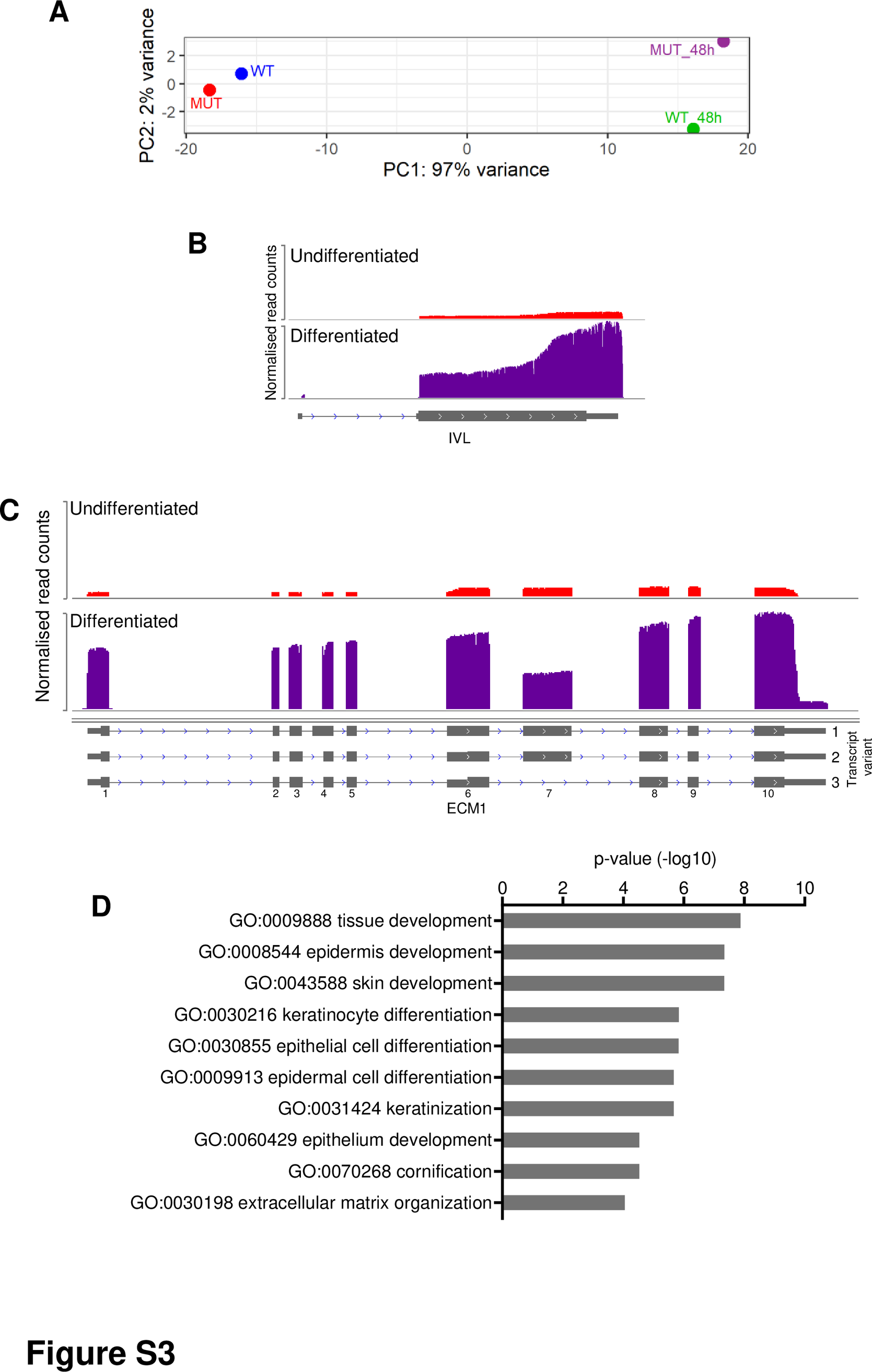

Supplement: S3 Fig — (A) PCA of host cell transcriptome in undifferentiated HFKs containing HPV18 (blue) and ΔCTCF-HPV18 (red) episomes and following 48hr incubation in methylcellulose (green and purple, respectively). Close clustering of HPV18 and ΔCTCF-HPV18 samples is observed in both undifferentiated and differentiated cell populations, indicating similar transcriptional profiles. Clear separation in PC1 is induced by host cell differentiation. (B-D) Gene expression changes in undifferentiated (red) and differentiated (purple) ΔCTCF-HPV18 genome-containing HFKs were analysed by long read Nanopore RNA-Seq, demonstrating enhanced involucrin (IVL) expression (B) and enhanced ECM1 expression combined with differentiation-induced exon 7 skipping in transcript variant 3; exon numbering and transcript variants are indicated to the right and below the ECM1 gene annotation (C). (D) Gene set enrichment analysis of differentiation-induced host differential gene expression in HPV18 and ΔCTCF-HPV18 episome containing HFKs. The top 10 most significant terms in Gene Ontology set; Biological Processes are shown with associated p value (-log10). (TIF) [file ppat.1010032.s003.tif]

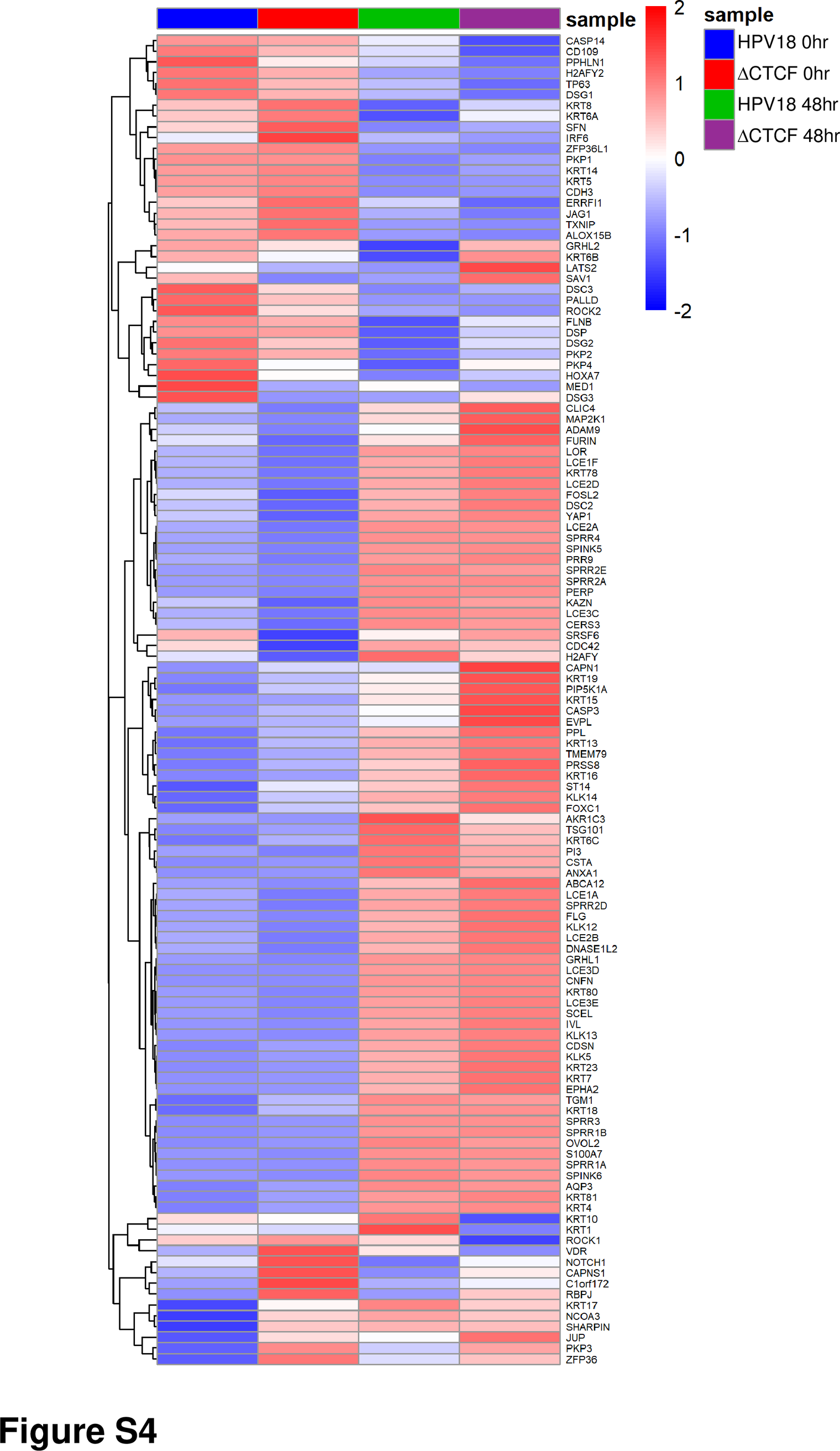

Supplement: S4 Fig — Heatmap showing differentiation-induced expression changes of genes within Biological Processes term GO:0030216:Keratinocyte Differentiation with a mean normalised count of >10 in HPV18 and ΔCTCF-HPV18 genome containing HFKs. (TIF) [file ppat.1010032.s004.tif]

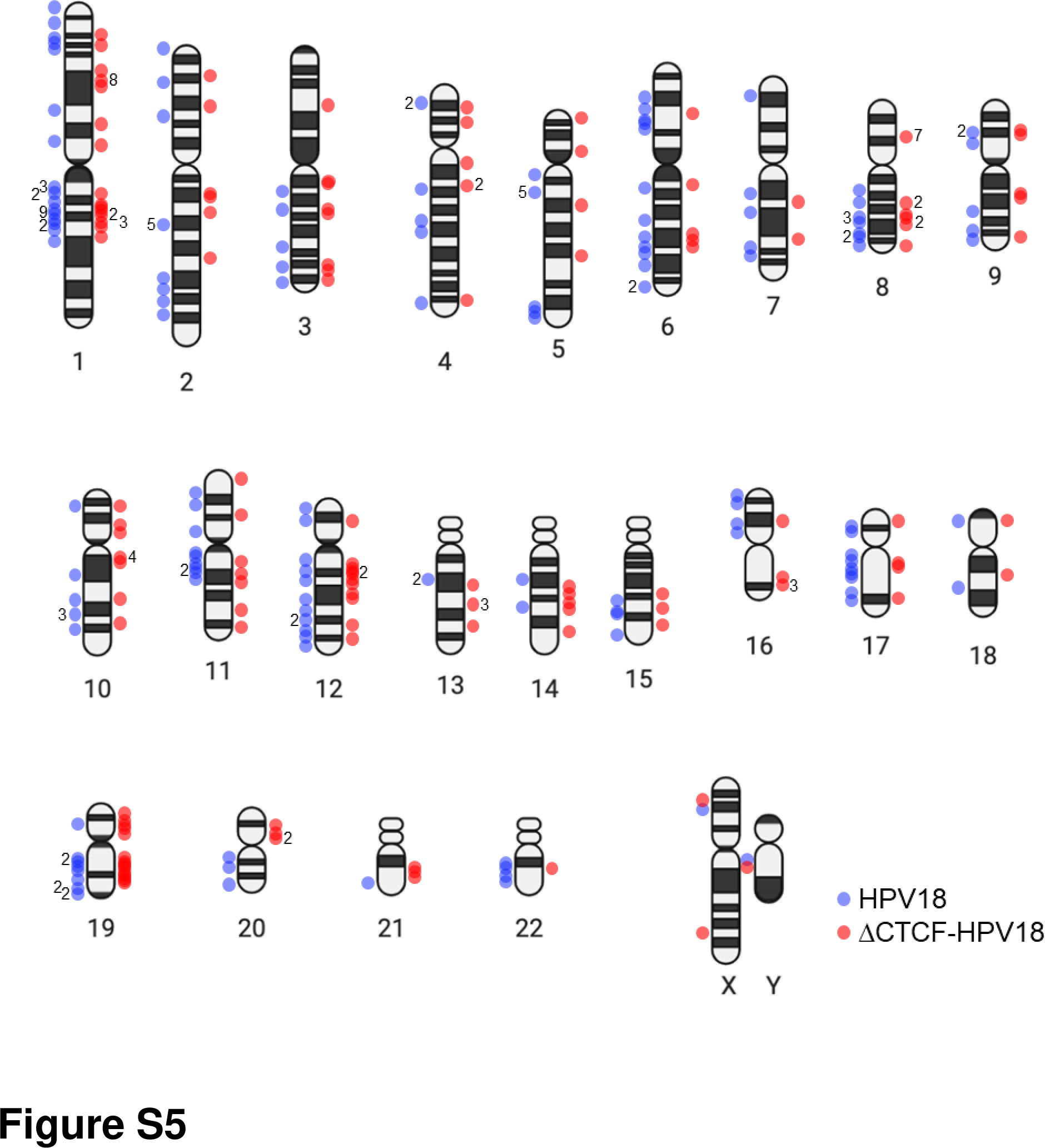

Supplement: S5 Fig — Approximate location of human-HPV fusion transcripts is highlighted on the karyotype (image from BioRender) for HPV18 (blue) and ΔCTCF-HPV18 (red). Where multiple transcripts with identical virus-host fusion co-ordinates were identified, the number of reads is indicated. (TIF) [file ppat.1010032.s005.tif]

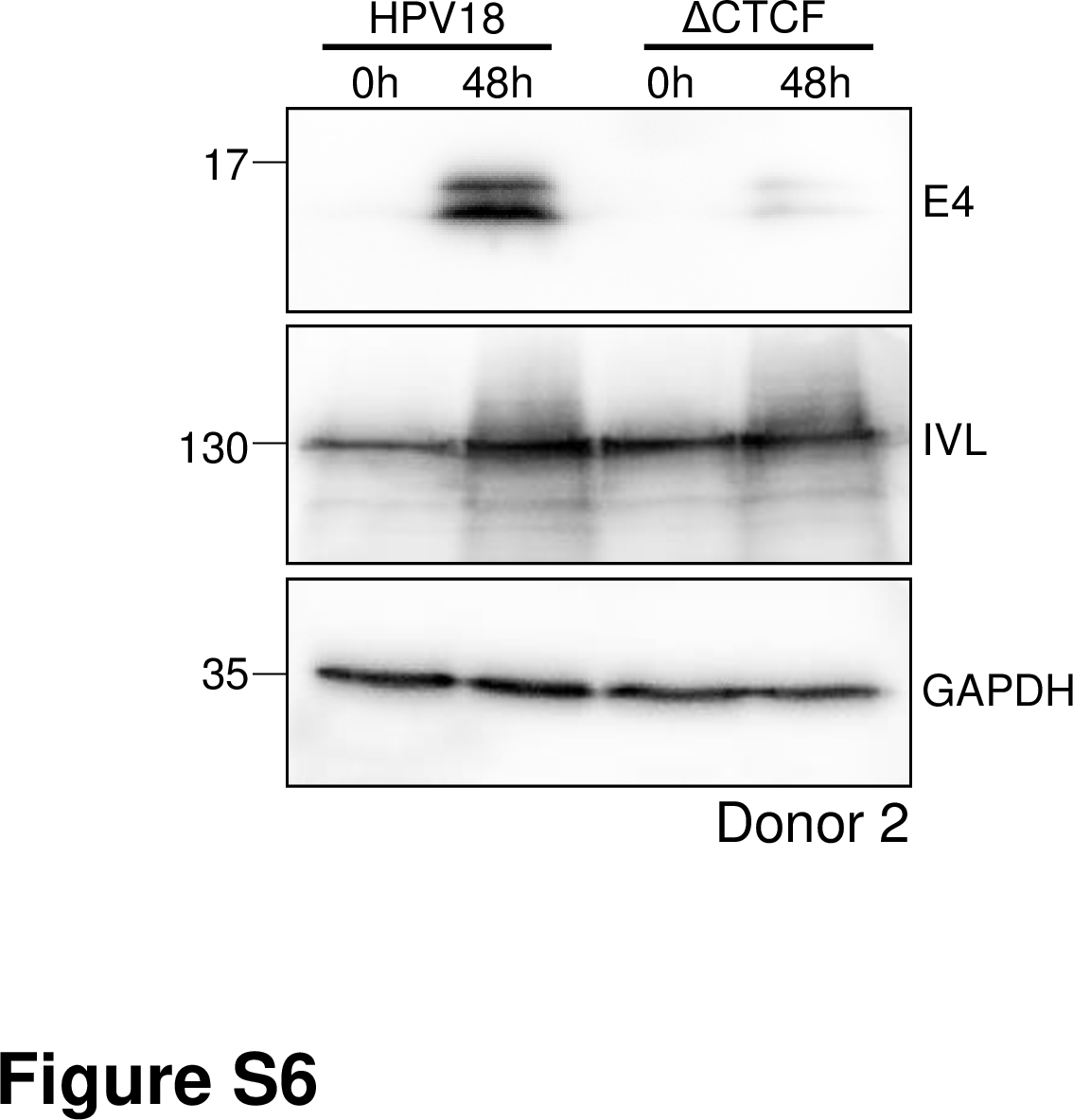

Supplement: S6 Fig — HPV18 genome-containing keratinocytes (Donor 2; HPV18 or ΔCTCF) grown in monolayer (undifferentiated, 0h) or differentiated in methylcellulose (48h) and E1^E4, involucrin (IVL) and GAPDH protein expression analysed by Western blotting. Molecular weight markers are indicated on the left (kDa). (TIF) [file ppat.1010032.s006.tif]
