# Supplementary material for: The chromatin insulator CTCF regulates HPV18 transcript splicing and differentiation-dependent late gene expression
Source: PLoS Pathog. 2021 Nov 4;17(11):e1010032. doi: 10.1371/journal.ppat.1010032 (PMC8594839; doi:10.1371/journal.ppat.1010032)
Supplement: S1 Table — Showing the nearest annotated human gene, coordinates of identified transcripts mapped to the human (Hg19) and HPV genomes and the number of reads detected. (DOCX) [file ppat.1010032.s007.docx]

| Nearest human gene | Human chr | Human gene coordinates | HPV18 coordinates | Read part mapping to human | Read part mapping to HPV18 | Number of reads detected |
| --- | --- | --- | --- | --- | --- | --- |
| PLEKHN1 | 1 | 901877-911245 | 830-929,3434-4267 | 991-2756 | 11-932 | 1 |
| CAPZB | 1 | 19665267-19812066 | 3432-4379 | 1110-2672 | 18-909 | 1 |
| ATPIF1 | 1 | 28562620-28573417 | 862-929,3434-4272 | 894-1058 | 30-863 | 1 |
| PTP4A2 | 1 | 32372022-32410457 | 777-929,3434-4274 | 1071-2546 | 2-982 | 1 |
| MRPS15 | 1 | 36921319-36930038 | 114-929,3434-4265 | 7-853 | 919-2556 | 1 |
| RPL5 | 1 | 93297582-93307481 | 824-929,3434-4273 | 3-1029 | 1120-2052 | 1 |
| RP4-663N10.1 | 1 | 115825655-115910693 | 829-929,3434-4201 | 10-985 | 1089-1944 | 1 |
| CRCT1 | 1 | 152486978-152488486 | 118-233,416-929,3434-4273;  819-929,3434-3934;  811-929,3434-3934 | 4-713;  661-1310;  697-1344 | 786-2233; 4-588;  3-617 | 3 |
| LCE3D | 1 | 152551857-152552980 | 116-233,416-929,2776-4261;  821-929,3434-4200 | 20-593;  76-611 | 677-2753  763-1610 | 2 |
| SPRR3 | 1 | 152974223-152976332 | 827-929,3434-4254 | 3-823 | 898-1790 | 1 |
| SPRR1B | 1 | 153003678-153005376 | 115-233,416-929,3434-4256 | 3-590 | 650-2097 | 1 |
| S100A9 | 1 | 153330330-153333503 | 117-233,416-929,3434-4273;  22-929,3434-4269;  880-929,3434-4253;  841-929,3434-4388;  114-233,416-929,3434-4264;  817-929,3434-4240;  829-929,3477-4273;  855-929,3434-4272;  774-929,3434-4264 | 1565-2093;  1867-2388;  943-1448;  21-1120;  14-481;  1009-1541;  907-1385;  1077-1583;  1201-1507 | 4-1477;  145-1802;  64-864;  612-1628;  614-2091;  4-901;  10-836;  49-985;  6-967 | 9 |
| S100A6 | 1 | 153507075-153508720 | 825-929,3434-4269 | 11-413 | 487-1414 | 1 |
| S100A14 | 1 | 153586731-153589462 | 820-929,3434-4388;  824-929,3434-4258 | 1157-2122;  1017-2028 | 8-1083;  12-932 | 2 |
| LINC01133 | 1 | 159931008-159948851 | 826-929,3434-4254 | 6-922 | 1008-1917 | 1 |
| PRDX6 | 1 | 173446405-173457946 | 845-929,3434-4248 | 987-1864 | 9-899 | 1 |
| RPS7 | 2 | 3622795-3628509 | 115-929,3434-4314 | 10-634 | 707-2381 | 1 |
| HADHA | 2 | 26413504-26467594 | 838-929,3434-4273 | 1513-4046 | 9-900 | 1 |
| RTN4 | 2 | 55199325-55339757 | 139-233,416-974 | 688-1583 | 26-679 | 1 |
| AC078882.1 | 2 | 142888747-142895399 | 118-929;  116-233,416-929;  116-233,416-929;  116-233,416-929;  113-233,416-929; | 797-1875;  603-1646;  618-1690;  624-1745;  654-1713 | 7-798;  3-604;  8-619  4-623;  1-626 | 5 |
| HSPE1-MOB4 | 2 | 198365137-198415450 | 824-929,3434-4273 | 1077-1546 | 4-948 | 1 |
| SGOL2 | 2 | 201374731-201448505 | 856-929,3465-4253 | 887-2259 | 1-815 | 1 |
| EEF1B2 | 2 | 207024309-207027652 | 851-929,3434-4273 | 1-789 | 853-1792 | 1 |
| RPL37A | 2 | 217362912-217443903 | 838-929,3506-4265 | 946-1259 | 22-848 | 1 |
| RP11-180K7.1 | 3 | 112521325-112531666 | 117-233,416-929 | 44-555 | 557-1183 | 1 |
| CNBP | 3 | 128888327-128902765 | 825-929,3434-4254 | 1014-2566 | 41-944 | 1 |
| RARRES1 | 3 | 158414681-158450485 | 824-929,3434-4272 | 1029-2045 | 61-947 | 1 |
| CLDN1 | 3 | 190023490-190040264 | 817-929,3434-4269 | 1066-2243 | 64-998 | 1 |
| RPL35A | 3 | 197676858-197683481 | 837-929,3434-4273 | 1032-1372 | 4-936 | 1 |
| S100P | 4 | 6694796-6698897 | 872-929,3434-4273;  1-233,416-929,3434-4273 | 913-1345;  4-102 | 4-857;  612-2181 | 2 |
| AC112518.3 | 4 | 74576019-74580244 | 778-929 | 79-337 | 336-492 | 1 |
| ANK2 | 4 | 113739265-114304896 | 813-929,3434-4259 | 73-489 | 531-1457 | 1 |
| USP53 | 4 | 120133742-120216672 | 745-929,3434-4272 | 10-1585 | 1650-2684 | 1 |
| SLC25A4 | 4 | 186064395-186071536 | 822-929,3434-4257 | 90-406 | 546-1433 | 1 |
| RP11-478P10.1 | 5 | 57186154-57194989 | 121-233,416-2455 | 14-693 | 690-2866 | 1 |
| Y_RNA | 5 | 64419196-64419301 | 114-930;  116-233,416-928;  418-930;  114-233,416-930;  116-233,416-928 | 835-2222;  642-926;  566-2786;  651-1974;  641-2012 | 1-837;  3-641;  47-568;  5-646;  7-633 | 5 |
| PRELID1 | 5 | 176730775-176733960 | 828-929,3434-4254 | 2-1231 | 1299-2206 | 1 |
| SQSTM1 | 5 | 179233388-179265078 | 826-929,3434-4272 | 24-1358 | 2075-2976 | 1 |
| GNB2L1 | 5 | 180663909-180675096 | 3539-4258 | 845-1886 | 10-761 | 1 |
| RP1-209A6.1 | 6 | 22744624-23032009 | 1213-1357,3434-3617 | 341-1023 | 10-312 | 1 |
| NEU1 | 6 | 31825436-31830683 | 825-929,3434-4273 | 1076-2797 | 50-981 | 1 |
| MAPK13 | 6 | 36095586-36107842 | 3993-4270 | 1268-3091 | 3168-3438 | 1 |
| RNF8 | 6 | 37321748-37362514 | 3663-4272 | 6-712 | 772-1366 | 1 |
| CCDC167 | 6 | 37450696-37467698 | 117-233,416-929,3434-4273 | 1546-2103 | 8-1482 | 1 |
| EEF1A1 | 6 | 74225473-74233520 | 840-929,3434-4261 | 9-1734 | 1816-2752 | 1 |
| SNX3 | 6 | 108532426-108582464 | 843-929,3434-4273 | 997-2122 | 6-915 | 1 |
| SERINC1 | 6 | 122764499-122792967 | 836-929,3434-4264 | 13-3109 | 3180-4112 | 1 |
| RPS12 | 6 | 133135580-133138703 | 824-929,3434-4252 | 1009-1501 | 5-937 | 1 |
| HEBP2 | 6 | 138724668-138743334 | 817-929,3434-4257 | 4-784 | 872-1805 | 1 |
| AL590867.1 | 6 | 153552455-153668623 | 820-929,3434-4385 | 4-394 | 556-1613 | 1 |
| PSMB1 | 6 | 170844205-170862429 | 35-233,416-929,3434-4273;  827-929,3434-4266 | 64-758;  63-890 | 961-2538;  978-1853 | 2 |
| NDUFA4 | 7 | 10971578-10979883 | 22-233,416-929,3434-4314 | 1752-2205 | 7-1567 | 1 |
| HSPB1 | 7 | 75931861-75933612 | 826-929,3434-4273 | 4-743 | 817-1746 | 1 |
| UPK3BL | 7 | 102277472-102283238 | 3527-4317 | 988-1535 | 11-741 | 1 |
| DGKI | 7 | 137065783-137531838 | 3966-4253 | 11-317 | 429-708 | 1 |
| TMEM178B | 7 | 140774032-141180180 | 114-233 | 10-617 | 618-736 | 1 |
| C8orf59 | 8 | 86126311-86132650 | 822-929,3434-4271 | 980-1382 | 7-907 | 1 |
| RPL30 | 8 | 99037079-99058697 | 812-929,3434-4255 | 1082-1500 | 64-1016 | 1 |
| TRPS1 | 8 | 116420724-116821899 | 123-233,416-929;  117-233,416-918;  425-921 | 6-1288;  8-580;  64-663 | 1287-1897;  589-1219;  685-1170 | 3 |
| UTP23 | 8 | 117778742-117861702 | 812-929,3434-4273 | 1103-3390 | 7-926 | 1 |
| RP11-398G24.2 | 8 | 122966879-123139423 | 114-233,413-929 | 629-980 | 2-630 | 1 |
| RP11-382A18.2 | 8 | 128351519-128404876 | 116-233,416-929;  117-926 | 620-747; 795-915 | 3-621;  1-789 | 2 |
| PTK2 | 8 | 141667999-142012315 | 116-233,416-929 | 635-769 | 2-636 | 1 |
| CDKN2B | 9 | 22002902-22009362 | 821-929,3434-4260;  135-233,416-929,3434-4257 | 41-845;  1495-3598 | 900-1777;  8-1428 | 2 |
| AQP3 | 9 | 33441152-33447609 | 3694-4273 | 670-1195 | 5-592 | 1 |
| TSTD2 | 9 | 100362362-100395962 | 866-929,3434-4254 | 1857-3352 | 63-910 | 1 |
| LCN2 | 9 | 130911350-130915734 | 3762-4252 | 5-787 | 866-1331 | 1 |
| EEF1A1P5 | 9 | 135894816-135896553 | 117-233,3434-4262 | 2-548 | 637-1580 | 1 |
| AKR1C3 | 10 | 5077546-5149878 | 838-929,3434-4388 | 1107-2294 | 5-1037 | 1 |
| ANK3 | 10 | 61786056-62493248 | 778-929,3434-4312 | 1102-1486 | 4-1025 | 1 |
| BLNK | 10 | 97951458-98031344 | 841-929,3434-4273 | 1014-2234 | 7-928 | 1 |
| RP11-446H13.2 | 10 | 108309864-108310879 | 117-233,416-929;  499-929,3434-3681;  114-233,416-1272 | 632-1043;  689-1113;  3-520 | 6-630;  4-687;  539-1496 | 3 |
| RP11-572P18.1 | 10 | 122114177-122114718 | 824-929,3434-4266 | 3-510 | 591-1487 | 1 |
| SWAP70 | 11 | 9685624-9774538 | 3519-4254 | 2-385 | 598-1215 | 1 |
| PLEKHA7 | 11 | 16799842-17035990 | 734-929,3434-4272 | 58-441 | 510-1542 | 1 |
| MTCH2 | 11 | 47638867-47664175 | 825-929,3434-4253 | 31-1043 | 1132-2054 | 1 |
| ZFP91-CNTF | 11 | 58346645-58392112 | 824-929,3434-4273 | 1035-3499 | 5-958 | 1 |
| STX3 | 11 | 59480929-59573354 | 830-929,3434-4254 | 1063-1688 | 72-995 | 1 |
| CPSF7 | 11 | 61170121-61197503 | 116-233,416-929,3434-4266 | 8-290 | 346-1838 | 1 |
| FTH1 | 11 | 61727190-61735132 | 3805-4264  844-929,3434-4273 | 526-1385  980-1215 | 15-446  11-922 | 2 |
| SLC3A2 | 11 | 62623518-62656352 | 833-929,3434-4273 | 4-1762 | 1851-2749 | 1 |
| FAU | 11 | 64888100-64889945 | 828-929,3434-4268 | 12-499 | 579-1554 | 1 |
| TSPAN9 | 12 | 3186521-3395730 | 846-929,3434-4273 | 1092-1421 | 59-1002 | 1 |
| GAPDH | 12 | 6643093-6647537 | 841-929,3434-4253 | 1087-2229 | 3-944 | 1 |
| MGST1 | 12 | 16500076-16762193 | 118-233,416-776,5211-6006 | 5-1377 | 1091-2339 | 1 |
| TMEM106C | 12 | 48357352-48362661 | 4075-4266 | 18-1334 | 1408-1587 | 1 |
| TMBIM6 | 12 | 50101508-50158717 | 828-929,3434-4273 | 14-1019 | 377-1278 | 1 |
| KRT6A | 12 | 52880958-52887041 | 779-929,3434-3647 | 7-475 | 496-854 | 1 |
| RP11-511B23.2 | 12 | 93397191-93609455 | 814-929,3434-4264 | 1059-1458 | 60-973 | 1 |
| METAP2 | 12 | 95867296-95909615 | 839-929,3434-4267 | 5-1874 | 1950-2869 | 1 |
| TMPO | 12 | 98909290-98944157 | 821-929,3434-4258 | 987-1351 | 1-904 | 1 |
| RPL18AP3 | 12 | 104659056-104659669 | 777-929,3434-4273  811-929,3434-4273 | 3-613  1043-1636 | 664-1683  2-976 | 2 |
| RPL6 | 12 | 112842994-112856642 | 821-929,3434-4300 | 1110-1976 | 82-1041 | 1 |
| RPLP0 | 12 | 120634489-120639038 | 3968-4273 | 7-752 | 820-1133 | 1 |
| CDK2AP1 | 12 | 123745528-123756881 | 826-929,3434-4273 | 67-1309 | 1385-2336 | 1 |
| TPT1 | 13 | 45911008-45915505 | 785-929,3434-4272;  3730-4262 | 16-1120;  664-1452 | 1225-2194;  59-589 | 2 |
| RPS29 | 14 | 50043390-50065408 | 812-929,3434-4262 | 1081-1341 | 72-982 | 1 |
| LIN52 | 14 | 74551499-74667936 | 113-233,416-929 | 641-2895 | 3-637 | 1 |
| SERF2 | 15 | 44069285-44094787 | 853-929,3434-4273 | 946-1445 | 1-884 | 1 |
| PKM | 15 | 72491370-72524164 | 810-929,3434-4260 | 12-2257 | 2340-3283 | 1 |
| MORF4L1 | 15 | 79102829-79190475 | 839-929,3434-4273 | 9-385 | 476-1379 | 1 |
| RP11-66B24.4 | 15 | 101433945-101459488 | 821-929,3434-4266 | 7-1005 | 1086-1982 | 1 |
| ATP6C | 16 | 2563966-2577956 | 821-929,3434-4272 | 7-933 | 992-1930 | 1 |
| PPL | 16 | 4932508-5010742 | 812-929,3434-4272 | 1029-2684 | 4-963 | 1 |
| RPS15A | 16 | 18792617-18801705 | 811-929,3434-4225 | 1201-1651 | 93-982 | 1 |
| PYCARD | 16 | 31212806-31214771 | 845-929,3434-4273 | 14-670 | 737-1541 | 1 |
| TRAPPC1 | 17 | 7833663-7835441 | 828-929,3434-4273 | 1004-1760 | 5-933 | 1 |
| FAM211A | 17 | 16344891-16395467 | 747-929,3434-4272 | 1089-1489 | 3-1020 | 1 |
| AC010761.8 | 17 | 27048505-27049895 | 3432-4260 | 979-1142 | 77-909 | 1 |
| ERAL1 | 17 | 27181956-27188085 | 768-929,3434-4257 | 1047-2855 | 26-983 | 1 |
| RPL23 | 17 | 37004118-37010096 | 527-929,3434-4273 | 1318-1802 | 9-1236 | 1 |
| JUP | 17 | 39775692-39943183 | 3874-4264 | 566-3625 | 85-479 | 1 |
| RAB5C | 17 | 40276994-40307035 | 817-929,3434-4263 | 64-1504 | 1652-2523 | 1 |
| RNU6-305P | 17 | 69307309-69307383 | 110-233,416-929 | 56-495 | 512-1127 | 1 |
| JMJD6 | 17 | 74708919-74722866 | 313-512 | 219-1105 | 22-216 | 1 |
| NDC80 | 18 | 2571510-2616634 | 838-929,3434-4182 | 997-2845 | 1-841 | 1 |
| SERPINB7 | 18 | 61420169-61472604 | 885-929,3434-4273 | 36-1851 | 1969-2806 | 1 |
| RPL18A | 19 | 17970685-17974962 | 821-929,3434-4273 | 8-612 | 695-1614 | 1 |
| LGALS7B | 19 | 39279851-39282389 | 841-929,3434-4257;  774-929,3434-4273 | 27-442;  1096-1532 | 531-1434;  3-986 | 2 |
| RPS16 | 19 | 39923847-39926588 | 813-929,3434-4245 | 81-230 | 333-1229 | 1 |
| DMRTC2 | 19 | 42348806-42356401 | 839-929,3434-4253 | 805-1406 | 2-867 | 1 |
| LYPD3 | 19 | 43964939-43969812 | 4051-4262 | 52-1606 | 1685-1881 | 1 |
| RPS11 | 19 | 49999622-50002946 | 840-929,3465-4317  827-929,3434-4316 | 1054-1600  1056-1594 | 2-964  7-997 | 2 |
| AP2A1 | 19 | 50270225-50310370 | 1052-3618 | 1-587 | 603-3105 | 1 |
| RPS9 | 19 | 54704610-54752862 | 828-929,3434-4253  3713-4273 | 22-669  609-1264 | 756-1685  4-530 | 2 |
| RPS5 | 19 | 58897767-58906173 | 825-929,3434-4272  821-929,3434-4273 | 1041-1769  1087-1742 | 3-954  2-943 | 2 |
| ID1 | 20 | 30193086-30194318 | 842-929,3434-4210 | 6-1691 | 1005-1884 | 1 |
| CTSA | 20 | 44518783-44527459 | 825-929,3434-4269 | 1000-2821 | 3-930 | 1 |
| RPS21 | 20 | 60962172-60963576 | 846-929,3434-4266 | 5-234 | 285-1210 | 1 |
| CSTB | 21 | 45192393-45196326 | 814-929,3434-4254 | 1022-1617 | 8-930 | 1 |
| DRG1 | 22 | 31795509-31924726 | 880-929,3434-4257 | 958-1566 | 24-881 | 1 |
| H1F0 | 22 | 38201114-38203442 | 811-929,3434-4273 | 5-1020 | 1088-2040 | 1 |
| MAFF | 22 | 38597889-38612518 | 817-929,3434-4272 | 3-1129 | 1193-2121 | 1 |
| NHP2L1 | 22 | 42069934-42086508 | 826-929 | 72-451 | 450-554 | 1 |
| APOO | X | 23851470-23926057 | 827-929,3434-4317 | 4-679 | 786-1783 | 1 |
| TTTY14 | Y | 21034387-21239302 | 824-929,3434-4259 | 12-2017 | 2105-3002 | 1 |
|  |  |  |  |  | **TOTAL** | **175** |
