# Supplementary material for: The chromatin insulator CTCF regulates HPV18 transcript splicing and differentiation-dependent late gene expression
Source: PLoS Pathog. 2021 Nov 4;17(11):e1010032. doi: 10.1371/journal.ppat.1010032 (PMC8594839; doi:10.1371/journal.ppat.1010032)
Supplement: S2 Table — Showing the nearest annotated human gene, coordinates of identified transcripts mapped to the human (Hg19) and HPV genomes and the number of reads detected. (DOCX) [file ppat.1010032.s008.docx]

| Nearest human gene | Human chr | Human gene coordinates | HPV18 coordinates | Read part mapping to human | Read part mapping to HPV18 | Number of reads detected |
| --- | --- | --- | --- | --- | --- | --- |
| RPL11 | 1 | 24018269-24022915 | 821-929,3434-4264 | 1002-1548 | 2-918 | 1 |
| SERINC2 | 1 | 31882412-31907525 | 3971-4264 | 6-427 | 492-759 | 1 |
| RNF11 | 1 | 51701943-51739127 | 829-929,3434-4273 | 1029-1233 | 9-967 | 1 |
| FGGY | 1 | 59762310-60233347 | 114-233,416-929;  117-929;  114-233,416-929;  122-233,416-929;  117-233,416-929;  114-233,416-929;  116-233,416-929;  173-233,416-929 | 3-569;  9-175;  64-225;  74-227;  11-166;  62-219;  5-156;  13-172 | 568-1198;  174-963;  224-844;  226-835;  165-794;  218-826;  155-774; 171-758 | 8 |
| LEPROT | 1 | 65886270-65901690 | 4059-4272 | 284-1258 | 4-202 | 1 |
| LINC01057 | 1 | 95104017-95285837 | 116-233,416-929,3434-3717 | 961-3750 | 5-918 | 1 |
| NRAS | 1 | 115247090-115259515 | 109-233,416-929,3434-4264 | 5-1214 | 1297-2713 | 1 |
| MRPS21 | 1 | 150266289-150281414 | 3879-4269 | 454-868 | 2-368 | 1 |
| S100A10 | 1 | 151955391-151966866 | 758-929,3434-4262 | 1094-1690 | 13-1009 | 1 |
| CRCT1 | 1 | 152486978-152488486 | 818-929,3434-4267 | 968-1688 | 3-914 | 1 |
| LCE3E | 1 | 152538130-152539248 | 846-929,3434-4273 | 7-552 | 674-1576 | 1 |
| SPRR1B | 1 | 153003678-153005376 | 840-929,3434-4272 | 986-1503 | 3-878 | 1 |
| S100A9 | 1 | 153330330-153333503 | 820-929,3434-4273;  119-233,416-929,3434-4266 | 1007-1514;  22-515 | 10-915;  606-2014 | 2 |
| S100A8 | 1 | 153362508-153363664 | 841-929,3434-4273;  24-233,416-929,3434-4208;  824-929,3434-4266 | 982-1328;  2309-2667;  1050-1247 | 6-903;  55-1563;  12-887 | 3 |
| TMCO1 | 1 | 165696032-165796992 | 118-215,416-757 | 443-2062 | 2-386 | 1 |
| RABGAP1L | 1 | 174128548-174964445 | 116-233,416-929,3434-3760 | 6-870 | 877-1821 | 1 |
| CENPA | 2 | 26987157-27023935 | 3660-4273 | 673-1514 | 5-594 | 1 |
| PNPT1 | 2 | 55861400-55921045 | 1595-2131 | 521-2384 | 3-540 | 1 |
| LIMS3 | 2 | 110656005-110677180 | 123-929,3434-3745 | 1106-3445 | 10-1094 | 1 |
| LIMS3L | 2 | 111222628-111230511 | 118-233,416-929,3434-3745 | 21-2447 | 2460-3342 | 1 |
| DBI | 2 | 120124497-120130126 | 118-233,416-929 | 8-461 | 526-1141 | 1 |
| MIR4444-2 | 2 | 178077454 -178077527 | 849-929,3434-4253 | 3-1496 | 1574-2455 | 1 |
| CDCP1 | 3 | 45123770-45187914 | 3633-4254 | 631-3568 | 10-621 | 1 |
| SENP7 | 3 | 101043049-101232085 | 829-929,3434-4273 | 610-1534 | 5-915 | 1 |
| RPL24 | 3 | 101399935-101405626 | 833-929,3434-4254 | 961-1203 | 5-891 | 1 |
| ATP2C1 | 3 | 130569439-130735556 | 123-233,416-929,3434-4285 | 1558-5031 | 12-1469 | 1 |
| CPNE4 | 3 | 131252399-132004254 | 595-929 | 8-139 | 138-469 | 1 |
| AP2M1 | 3 | 183892477-183901879 | 827-929,3434-4272 | 967-2804 | 5-897 | 1 |
| LEPREL1 | 3 | 189674517-189840226 | 4035-4258 | 65-3213 | 3311-3526 | 1 |
| KIAA0226 | 3 | 197398264-197476598 | 824-929,3434-4273 | 4-1286 | 1351-2260 | 1 |
| S100P | 4 | 6694796-6698897 | 770-929,3434-4263 | 4-449 | 523-1487 | 1 |
| PI4K2B | 4 | 25162263-25280714 | 233-487 | 11-1170 | 1178-1418 | 1 |
| TMEM165 | 4 | 56262124-56319564 | 811-929,3434-4266 | 6-1841 | 1925-2866 | 1 |
| CXCL3 | 4 | 74902306-74904524 | 128-233,416-931;  113-233,416-922 | 588-1664;  614-1691 | 19-592;  2-606 | 2 |
| RWDD4 | 4 | 184560788-184580378 | 116-233,416-931 | 7-688 | 685-1303 | 1 |
| PDCD6 | 5 | 271736-353971 | 4052-4272 | 296-1309 | 24-237 | 1 |
| RPL37 | 5 | 40825364-40835437 | 825-929,3434-4273 | 60-404 | 497-1410 | 1 |
| COX7C | 5 | 85913721-85916779 | 117-233,416-929,3434-4271 | 3-407 | 487-1929 | 1 |
| EGR1 | 5 | 137801179-137805004 | 112-233,416-929,3434-4254 | 1-2823 | 2899-4293 | 1 |
| HIST1H2BD | 6 | 26158349-26171577 | 140-929,3434-4254 | 3-284 | 288-1866 | 1 |
| EEF1A1 | 6 | 74225473-74233520 | 3980-4273 | 395-2098 | 37-310 | 1 |
| RPS12 | 6 | 133135580-133138703 | 817-929,3434-4273 | 3-484 | 528-1435 | 1 |
| PERP | 6 | 138409642-138428648 | 839-929,3434-4388 | 1183-3056 | 62-1116 | 1 |
| ABRACL | 6 | 139349819-139364439 | 3896-4273 | 436-1214 | 5-360 | 1 |
| AZGP1 | 7 | 99564343-99573780 | 827-929,3434-4272 | 8-1151 | 1221-2118 | 1 |
| C7orf73 | 7 | 135347244-135378166 | 117-4262 | 4158-4435 | 4-4094 | 1 |
| DPYSL2 | 8 | 26371791-26515694 | 117-233,416-1509;  124-233,416-1509;  116-233,416-1509;  111-233,413-1503;  114-233,416-1509;  118-233,416-1509;  113-233,416-1509 | 1235-1385;  1192-1352;  1240-1399;  1179-1329;  1195-1322;  1156-1315;  1185-1337 | 1-1234;  11-1191;  5-1239;  3-1175;  4-1174;  5-1155  2-1184 | 7 |
| RP11-255L13.1 | 8 | 90255630-90256022 | 123-233,416-926  117-233,416-1935 | 5-887;  12-909 | 892-1519;  919-2520 | 2 |
| COX6C | 8 | 100885428-100906290 | 3952-4314 | 497-865 | 7-364 | 1 |
| YWHAZ | 8 | 101928753-101965616 | 811-929,3465-4388 | 63-1008 | 1054-2069 | 1 |
| ATP6V1C1 | 8 | 104033291-104085279 | 116-233,414-929;  116-233 | 13-293;  71-496 | 291-909  495-620 | 2 |
| LYPD2 | 8 | 143831568-143833952 | 77-233,416-929,3434-4244 | 956-2119 | 83-1555 | 1 |
| PLIN2 | 9 | 19108373-19149288 | 3432-4312 | 1329-1658 | 5-869 | 1 |
| RPS6 | 9 | 19375713-19380252 | 846-929,3434-4272 | 67-806 | 903-1811 | 1 |
| RPL35AP21 | 9 | 74204517-74204849 | 825-929,3434-4211 | 7-393 | 487-1341 | 1 |
| ANXA1 | 9 | 75766673-75785309 | 835-929,3434-4273 | 34-1341 | 1409-2316 | 1 |
| RPL12 | 9 | 130209953-130213684 | 839-929,3434-4259 | 7-614 | 683-1557 | 1 |
| LINC00704 | 10 | 4692377-4720346 | 131-233,416-929 | 6-400 | 432-1037 | 1 |
| RAB18 | 10 | 27793197-27831143 | 3433-4273 | 902-2899 | 3-830 | 1 |
| PARD3 | 10 | 34398488-35104253 | 233-967 | 764-1493 | 44-755 | 1 |
| ERCC6 | 10 | 50663414-50747584 | 113-233,416-929;  1046-1409;  1046-1409;  110-233,416-1409 | 633-1077; 365-2665;  419-2769;  1077-2345 | 2-631;  7-359;  52-408;  2-1066 | 4 |
| ERCC6-PGBD3 | 10 | 50723247-50747584 | 416-930 | 625-906 | 117-622 | 1 |
| ANKRD22 | 10 | 90581889-90611575 | 835-929,3434-4273 | 1018-2248 | 6-936 | 1 |
| RP11-572P18.1 | 10 | 122114177-122114718 | 778-929,3434-4266 | 42-555 | 618-1585 | 1 |
| POLR2L | 11 | 837356-842545 | 795-929,3434-4261 | 54-399 | 489-1397 | 1 |
| CD59 | 11 | 33719807-33757991 | 860-929,3434-4255 | 999-2549 | 14-891 | 1 |
| FTH1 | 11 | 61727190-61735132 | 74-233,3434-4266 | 1037-1902 | 9-971 | 1 |
| GSTP1 | 11 | 67351066-67354131 | 122-233,416-929,3434-4253 | 17-699 | 778-2147 | 1 |
| RP11-864N7.2 | 11 | 74456761-74457159 | 774-929,3434-4273 | 9-230 | 289-1271 | 1 |
| IL18 | 11 | 112013974-112034840 | 1435-1440,3543-4273 | 816-1868 | 5-873 | 1 |
| NTM | 11 | 131240373-132206716 | 117-233,416-929 | 65-755 | 764-1384 | 1 |
| MGST1 | 12 | 16500076-16762193 | 4001-4272 | 282-618 | 9-262 | 1 |
| KRT7 | 12 | 52626304-52645970 | 828-929,3434-4317 | 3-684 | 749-1734 | 1 |
| KRT81 | 12 | 52679697-52685318 | 817-929,3434-4257 | 993-2015 | 18-917 | 1 |
| KRT6B | 12 | 52840435-52845910 | 4786-7239;  841-929,3434-4263 | 5-2212;  950-3088 | 2325-4585;  9-893 | 2 |
| KRT5 | 12 | 52908359-52914471 | 117-233,416-804 | 733-2880 | 163-659 | 1 |
| CALCOCO1 | 12 | 54104903-54121529 | 3450-4255 | 834-1451 | 11-813 | 1 |
| PA2G4 | 12 | 56498103-56507691 | 116-929,2779-4254 | 2375-3922 | 2-2313 | 1 |
| RP11-230G5.2 | 12 | 65860600-66036152 | 460-929,3434-3696 | 712-1868 | 1-713 | 1 |
| RPSAP52 | 12 | 66151800-66220754 | 118-233,416-907,7078-7093 | 9-1008 | 495-1651 | 1 |
| RPL18AP3 | 12 | 104659056-104659669 | 3969-4312 | 412-1000 | 7-334 | 1 |
| POP5 | 12 | 121016567-121019201 | 3697-4272 | 870-1669 | 4-482 | 1 |
| TPT1 | 13 | 45911008-45915505 | 821-929,3465-4266 | 1047-1838 | 76-981 | 1 |
| ITM2B | 13 | 48807294-48837063 | 842-929,3434-4273 | 979-1920 | 3-905 | 1 |
| MARK2P12 | 13 | 73981905-73982489 | 114-233,416-929,3434-3762;  3656-3779;  64-233,416-930 | 966-1274;  140-2137;  664-1062 | 4-940;  2-135;  2-666 | 3 |
| GPC6 | 13 | 93879095-95059655 | 116-233,416-929 | 4-236 | 235-840 | 1 |
| RPS29 | 14 | 50043390-50065408 | 361-929,3434-4223 | 1700-2369 | 44-1363 | 1 |
| C14orf166 | 14 | 52456193-52471420 | 3432-3935 | 3-914 | 991-1482 | 1 |
| LGALS3 | 14 | 55590828-55612126 | 3703-4272 | 697-1513 | 21-560 | 1 |
| VTI1B | 14 | 68113792-68141548 | 120-233,416-929,3434-4253 | 1578-1820 | 63-1517 | 1 |
| CPSF2 | 14 | 92588281-92630755 | 824-929,3434-4273 | 7-4817 | 4891-5812 | 1 |
| RPL4 | 15 | 66790355-66816870 | 116-233,416-929,3434-4262 | 6-629 | 690-2127 | 1 |
| RPLP1 | 15 | 69745123-69748255 | 509-929,3440-4273 | 4-482 | 549-1736 | 1 |
| RHCG | 15 | 89998680-90039844 | 117-233,416-929,3434-4273 | 56-1945 | 2009-3448 | 1 |
| PPL | 16 | 4932508-5010742 | 840-929,3434-4264 | 56-6120 | 6199-7067 | 1 |
| LCMT1 | 16 | 25123050-25189552 | 811-929,3434-4388 | 5-1311 | 1397-2428 | 1 |
| CMC2 | 16 | 81009698-81053875 | 860-929,3434-4213 | 948-1636 | 36-851 | 1 |
| KCNG4 | 16 | 84255823-84273356 | 114-233,416-929,3434-3606;  116-233,416-929,3434-3608; 827-929,3434-3621 | 817-2519;  798-2065;  295-2068 | 5-801;  4-772;  2-290 | 3 |
| PFN1 | 17 | 4848947-4852356 | 741-929,3434-4273 | 1100-1835 | 5-1017 | 1 |
| KRT16 | 17 | 39766030-39772151 | 853-929,3434-4264 | 4-2153 | 1277-2184 | 1 |
| EIF1 | 17 | 39845137-39848920 | 821-929,3434-4273 | 6-662 | 713-1669 | 1 |
| RPL38 | 17 | 72199721-72206794 | 838-929,3434-4273 | 7-328 | 400-1293 | 1 |
| MYL12B | 18 | 3261907-3278282 | 2784-4267 | 9-831 | 926-2362 | 1 |
| RPL17 | 18 | 47014851-47018906 | 821-929,3434-4319 | 62-604 | 675-1665 | 1 |
| RPL36 | 19 | 5674958-5691887 | 839-929,3434-4273 | 132-352 | 451-1381 | 1 |
| RPS28 | 19 | 8386042-8388224 | 3618-4272 | 705-855 | 4-624 | 1 |
| EIF3G | 19 | 10225693-10230596 | 828-929,3434-3934 | 691-1656 | 11-624 | 1 |
| DNAJB1 | 19 | 14625582-14640582 | 880-929,3434-4267 | 9-2173 | 2244-3075 | 1 |
| RP11-255H23.4 | 19 | 24000401-24057503 | 6633-7303 | 748-1701 | 4-681 | 1 |
| C19orf33 | 19 | 38794801-38795649 | 113-233,416-929,3434-4273 | 15-346 | 421-1849 | 1 |
| EIF3K | 19 | 39109735-39127595 | 4013-4263 | 332-1017 | 2-234 | 1 |
| LGALS7B | 19 | 39279851-39282389 | 538-929,3434-4273 | 1285-1644 | 10-1200 | 1 |
| RPS19 | 19 | 42363988-42376994 | 3845-4272 | 1-498 | 580-1002 | 1 |
| RABAC1 | 19 | 42460833-42463542 | 812-929,3434-4254 | 972-1616 | 5-906 | 1 |
| FTL | 19 | 49468558-49470135 | 824-929,3434-4264 | 1029-1835 | 4-954 | 1 |
| ATF5 | 19 | 50431959-50437192 | 813-929,3434-4266 | 1049-1334 | 1-918 | 1 |
| KLK6 | 19 | 51461887-51472929 | 824-929,3434-4273 | 1004-1741 | 5-927 | 1 |
| CTB-147C22.9 | 19 | 51471466-51515385 | 821-929,3434-4272 | 1006-1941 | 4-947 | 1 |
| KLK10 | 19 | 51515995-51523431 | 116-233,416-929,3434-4273 | 7-1327 | 1410-2827 | 1 |
| MGME1 | 20 | 17949556-17971765 | 122-233,416-929,3434-4257 | 1541-3197 | 6-1452 | 1 |
| C20orf187 | 20 | 11008408-11010014 | 636-929 | 36-473 | 482-761 | 1 |
| RP5-1100I6.1 | 20 | 24123226-24125022 | 122-233,416-938;  117-233,416-949 | 3-649;  59-716 | 658-1282;  714-1361 | 2 |
| AF131217.1 | 21 | 29811667-30047170 | 68-233,416-929 | 56-650 | 649-1342 | 1 |
| MRPS6 | 21 | 35445524-35515334 | 114-929,3434-4264 | 1698-2513 | 6-1622 | 1 |
| SETD4 | 21 | 37406839-37451687 | 71-233,416-929,3434-4254 | 9-1116 | 1184-2696 | 1 |
| SELM | 22 | 31500758-31516055 | 826-929,3434-4266 | 6-639 | 718-1590 | 1 |
| SYAP1 | X | 16737755-16783459 | 880-929,3434-3758 | 395-696 | 20-391 | 1 |
| RPL39 | X | 118920467-118925606 | 1223-1357,3434-4231 | 9-362 | 470-1348 | 1 |
| TTTY14 | Y | 21034387-21239302 | 3734-4264 | 4-2437 | 2074-2602 | 1 |
|  |  |  |  |  | **TOTAL** | **157** |
