# Supplementary material for: The chromatin insulator CTCF regulates HPV18 transcript splicing and differentiation-dependent late gene expression
Source: PLoS Pathog. 2021 Nov 4;17(11):e1010032. doi: 10.1371/journal.ppat.1010032 (PMC8594839; doi:10.1371/journal.ppat.1010032)
Supplement: Attachment — Submitted filename: PLoS Path response to reviewers.docx [file ppat.1010032.s009.docx]

**Part II – Major Issues: Key Experiments Required for Acceptance**

**Reviewer #1**: 1). CTCF was shown to interact by looping with YY1 in the URR. Does mutation of the YYI site in the LCR recapitulate mutation of the CTCF site in E2 on late splicing effects?

There are multiple YY1 binding sites in the URR of HPV18, which overlap with the binding sites of other transcriptional regulators. As such, any transcriptional differences observed in such an experiment would be difficult to interpret. Nonetheless, we previously reported that shRNA-mediated depletion of YY1 resulted in significantly reduced intrachromosomal interactions between the viral LCR and E2-ORF. This loss of chromatin loop interaction is co-incident with increased chromatin accessibility and increased E6/E7 expression levels [1]. These published data provide good evidence that CTCF-mediated HPV early gene repression occurs through interaction with YY1.

YY1 levels decrease upon differentiation so it is unclear how loss of CTCF looping affects late events. Could the effects on late splice site choice be mediated by altered levels of an early gene product that is affected by CTCF YY1 looping?

CTCF has pleotropic effects on gene expression including chromatin looping, regulation of epigenetic boundaries and regulation of gene splicing through roadblock repression of transcription machinery. In this study we show altered gene splicing of early gene transcripts produced from ΔCTCF-HPV18 episomes. This alteration in splicing is most profound in undifferentiated cells so the differentiation-induced reduction in YY1 expression is not relevant in this case. However, we cannot rule out that alterations in the expression of early gene products affect splice site choice in both early and late transcripts and/or activation of the late promoter upon cellular differentiation. This has now been noted in the discussion of our revised manuscript (lines 889 to 890).

2). Is the 50% reduction in E1^E4 transcripts in the CTCF mutant the result of altered differentiation dependent replication or changes in cell cycle?

We have included data showing differentiation-induced transcriptional changes to host genes associated with keratinocyte differentiation in both wild type and ΔCTCF-HPV18 HFKs which show little difference in host cell markers of methylcellulose-induced differentiation (New Figures S3 and S4). In addition, we previously reported that the cellular DNA replication and cycle markers, BrdU incorporation and Cyclin B1 respectively, were both markedly increased in rafts derived from HPV18 and ΔCTCF-HPV18 HFKs in comparison to HPV18 negative HFK rafts. While there were subtle differences in the levels of these markers in distinct compartments of the epithelia, increased cell proliferation was observed in both wild type and mutant HPV18 rafts. We also demonstrated no difference in both the growth kinetics of HPV18 and ΔCTCF-HPV18 HFKs grown in monolayer, and in phospho-H3 staining of raft sections, demonstrating no difference in mitotic entry in HPV18 and ΔCTCF-HPV18 rafts [2].

Southern analysis should be performed to look at genome levels upon differentiation with the CTCF mutant genomes.

We have now included Southern blot analysis of two independent HFK donor lines showing equivocal methylcellulose-induced amplification of wild type and mutant HPV18 episomes (New Figures 2A and S2).

Does the previously reported increase in E6/E7 transcription upon CTCF site mutation affect induction of late functions?

We agree that it is possible that increased E6/E7 expression could affect the induction of late gene expression. We have added discussion of this possibility to the revised manuscript (lines 889 to 890).

Is reduced E1^E4 responsible for altered splicing patterns? Is there a difference in how CTCF affects splice site choice in the early region as opposed to late?

Splicing differences in the early transcripts are observed in undifferentiated cells where E1^E4 expression is not expressed and therefore is not driving these differences. Furthermore, the full programme of late gene expression is not induced by methylcellulose differentiation so any effect on splicing of L1/L2 transcripts is not addressed in this study.

3). The reduction in H4Ac is interesting but the effects only reduce late promoter activity by 50%. Is there an increase in repressive histone marks or other modified histones on the late or early regions?

Our data show that methylcellulose differentiation of HPV18 WT HFKs results in a dramatic increase in H4Ac, particularly in the region of the early promoter. In contrast, although a small increase in H4Ac abundance is observed in ΔCTCF-HPV18 episomes upon differentiation, this is dramatically lower than that seen in WT. This correlates with the observed attenuation (not complete loss of activity) of late promoter activation upon differentiation of CTCF-binding defective episomes. We apologise that this point was not clear in the original submission and we edited our description of these data accordingly (lines 661-664).

In our previous study, we reported that repressive H3K27Me3 marks are highly enriched at the early promoter of WT HPV18 episomes in undifferentiated cells, which is in stark contrast to ΔCTCF-HPV18 episomes which have very low enrichment of H3K27Me3. Differentiation of HPV18 WT cells resulted in a dramatic loss of H3K27Me3, while no change was observed in ΔCTCF-HPV18 (H3K27Me3 was low and stayed low upon differentiation). We concluded that CTCF-YY1 mediated chromatin looping between E2-CTCF binding site and the LCR stabilises association of the PRC complex resulting in epigenetic transcription repression (enriched H3K27Me3). Differentiation disrupts the stability of the chromatin loop resulting in loss of epigenetic repression of virus transcription. While the differentiation dependent changes to H3K27Me3 enrichment correlated with early promoter activity in WT and ΔCTCF HPV18 episomes, we did not go on to determine whether there were CTCF-dependent changes to differentiation-induced late promoter activation. In the present study, we observed a significant reduction in transcripts originating from the late promoter and wanted to determine whether this is due to differential epigenetic activation of late transcription. H4Ac is a hallmark of enhanced activation of transcription by facilitating increased chromatin accessibility and the recruitment of transcriptional activators and we therefore studied the differentiation-dependent enrichment of this mark in WT and ΔCTCF-HPV18 episomes. The justification as to why we opted to study H4Ac above other histone marks is now included in our manuscript (lines 645 to 658).

**Reviewer #2:** 1. Nearly one-third of the abstract is reporting previously published results. The focus should be more on the current study, the results and the implications/significance.
Background information pertinent to our study was included in the abstract to set the scene for the present study. Nonetheless, we have trimmed this section of the abstract as suggested by the reviewer.

2. More details should be provided of the E2 ORF CTCF binding site. This should be given a precise location (other HPV genomic features are) rather than a generic location. A sequence and co-ordinates should be provided as well as an alignment with the CTCF core consensus motif.

Details of the mutations introduced in the CTCF binding in the HPV18 E2 ORF have been previously published and we therefore did not include these in the present manuscript. However, we agree that these important details should also be included here. We have therefore included a new figure (Fig.1A) which provides details of the core CTCF binding consensus sequence aligned with the HPV18 E2-ORF CTCF binding site and the conservative mutations that were introduced to abrogate CTCF binding.

3. ChIP sequencing nicely identified differences in binding to WT vs CTCFmutant HPV in infected keratinocytes. However, no changes in host CTCF genome-wide binding were mentioned. These should be analysed and described (at least in Supplementary data).

We did analyse differences in CTCF binding in two independent HFK donors harbouring either wild type HPV18 or ΔCTCF-HPV18 episomes and observed a high degree of overlap in host genome-wide CTCF enriched peaks. These data are now included as New Supplementary Figure S1.

4. In Figure 1B what differences in host gene expression was observed? If there were changes in gene expression in HPV genome due to the deletion of the CTCF sites, then changes in host gene expression would also be expected. Please detail.

Analysis of differential gene expression in our Illumina and MinION datasets reveals that there are only very subtle differences in host cell gene expression between wild type and ΔCTCF-HPV18 episome, as highlighted by the principal component analyses of our Illumina data (below) and our MinION data (New Fig.S3A) and the heatmap of differentiation-induced changes to gene set; Keratinocyte differentiation (New Fig.S4). We hypothesise that the ~2 fold increase in early transcript production in ΔCTCF-HPV18 episomes compared to wild type does not have any further notable effect on the host transcriptome as the viral oncogenes are known to be potent even when expressed at low level. However, since the differences in host gene expression can only be assessed in our single sample sequencing experiments, albeit with consistencies across the two different next generation sequencing methods used, it is not appropriate to draw firm conclusions regarding these differences without further validation.

**Figure**: Principal Component Analysis of top 500 differentially expressed genes in HPV18 (WT) and ΔCTCF-HPV18 (MUT) episome containing HFKs compared to untransfected (UT) HFKs. Data show HPV18-induced alteration of prinicipal component (PC)1, but little variance in PC2. HPV18 and ΔCTCF-HPV18 samples are closely clustered, indicating similar induction of differential gene expression in both datasets.

5. Examples of differentiation-dependent host cell gene expression in HPV-infected keratinocytes were provided (IVL and EMC1) but this was limited at best – cherry picking at worst. More genes need to be examined to create an overall picture. This could be in Supplementary.

We originally included differential expression analysis of two genes in our MinION datasets to demonstrate that incubation of our HPV18 HFKs in methylcellulose for 48hrs induced keratinocyte differentiation. Involucrin is a commonly used marker of keratinocyte differentiation (e.g.[2]), and ECM1 has been clearly shown to be differentially spliced during keratinocyte differentiation [3] and therefore served as an important control for the analysis of differentiation-induced differential splicing of viral transcripts. Nonetheless, as requested, we have now included a full analysis of differentiation-induced gene expression changes in wild type and ΔCTCF-HPV18 HFKs and have included the results of gene set enrichment analysis showing significant enrichment of genes associated with keratinocyte differentiation (Fig.S3D) and visualisation of differentiation-dependent gene expression changes as a heatmap (Fig.S4).

6. Line 315: ‘Viral host fusion transcripts’ – Is there evidence, if any, that an absence of CTCF binding site in HPV genome could affect HPV viral integration? Loss of a CTCF site could affect episomal folding and/or association with the host genome and/or viral integration. Please comment in Discussion.

Only a small proportion of virus-host fusion transcripts were identified in our MinION datasets, which implies a low frequency of transcriptionally active genome integration. Furthermore, there were no obvious differences between the frequency and location of HPV18 integration sites between wild type and ΔCTCF-HPV18 episomes (see New Tables S1 and S2 and new Figure S5), suggesting that CTCF binding at the E2 ORF does not influence HPV18 integration in our primary cell-based model of HPV replication. These findings have now been included in the results section of our revised manuscript (lines 415 to 417).

7. Why was H4 acetylation examined as opposed to other histone marks? This is not made clear.

We have previously reported that H3K4Me3, a hallmark of activated promoter and TSS regions, is detected at very low abundance at the HPV18 LCR and early gene region of HPV18 wild type episomes in undifferentiated HFKs. Differentiation of HFKs resulted in a significant increase in H3K4Me3, particularly at the P_811_ late promoter [1]. In the same study, we also showed that H3K4Me3 was detected at significantly higher abundance in ΔCTCF-HPV18 episomes than wild type in undifferentiated HFKS, correlating with increased early promoter activity and E6/E7 transcript abundance. However, in contrast to wild type HPV18, H3K4Me3 was not further increased in abundance at the late promoter upon cellular differentiation in ΔCTCF-HPV18 episomes; the distribution of the histone mark remained unchanged upon cellular differentiation. These findings suggested that the late promoter is not efficiently activated upon keratinocyte differentiation when CTCF binding is defective, but we did not go on to further investigate differentiation-dependent late promoter activation in our previous publication.

In the present study, we observed a significant reduction in transcripts derived from the P_811_ late promoter in our Nanopore analysis of differentiated ΔCTCF-HPV18 HFKs compared to wild type, which we opted to investigate further. While H3K4Me3 modifications are a hallmark of active promoter regions, downstream acetylation of lysines within histones 3 and 4 is highly correlated with high gene expression as acetylation of histone tails induces an open chromatin conformation and recruits bromodomain containing transcriptional regulators (reviewed in [4]). Although we previously observed enhanced H3K4Me3 abundance in ΔCTCF-HPV18 episomes, suggesting increased promoter activity (particularly the early promoter), the absence of H4Ac enrichment following host cell differentiation in ΔCTCF-HPV18 episomes we now show demonstrates that differentiation-induced increases in transcriptional activity do not occur when CTCF binding is abrogated.

The justification of this experiment and our interpretation of the data have now been more clearly discussed in the revised manuscript (lines 647 to 653).

8. The confirmation of differentially expressed HPV proteins by Western blot is a bit limited (Figure 7a & E) and only E1^E4 is shown. This reviewer would have more confidence in CTCF’s role in regulating late viral gene expression if more HPV proteins are examined. If possible, a viral protein that is predicted to remain unchanged (based on Nanopore sequencing) should also be included. E6 and E7 should be examined.

We previously showed that E2 protein expression was not altered in organotypic raft cultures derived from ΔCTCF-HPV18 genome-containing HFKs in comparison to wild type by both Western blotting and immunofluorescence staining [5]. We also showed by Western blotting that E6 and E7 protein levels are increased in undifferentiated cells grown in monolayer and in organotypic raft culture when the CTCF binding site is mutated [1, 5]. Nonetheless, we agree that these findings are important controls for the present study, and we have therefore included further Western blotting analysis of E2, E6 and E7 protein expression in organotypic raft culture lysates (Fig.7F and G). These new data show that E2 protein levels are unchanged, while E6 and E7 protein levels are increased in lysates extracted from three independent raft cultures, in agreement with our Illumina and Nanopore datasets.

9. Figure 7A: The authors had previously shown CTCF expression increase upon HPV transduction. However, this has not been yet shown for differentiation. For completeness, total CTCF protein levels should also be examined.

CTCF protein expression levels in differentiated keratinocytes were assessed in our previously published manuscript and compared to expression levels in undifferentiated donor matched keratinocytes ([1], see Fig.6C therein). These data showed no change in CTCF protein expression upon keratinocyte differentiation. We have now included discussion of these findings in the present manuscript (lines 109 to 110).

10. A mechanism by which CTCF regulates alternative splicing is via the ‘roadblock model’ by pausing RNAPII elongation. Is RNAPII enriched at the HPV CTCF site during keratinocyte differentiation?

We agree that ChIP analysis of RNAPolII enrichment would add mechanistic insight to how CTCF might influence HPV transcript splicing. We have therefore put significant effort into completing these experiments (despite Covid-19 enforced reduced laboratory access). Unfortunately, after trialling several different RNAPolII antibodies, we are still unable to reproducibly ChIP HPV episomes. We will continue to overcome these technical difficulties and aim to unravel the mechanism of CTCF-mediated HPV splicing control in the future.

11. Are cohesin subunits eg RAD21 enriched at the HPV CTCF site and enforcing the CTCF-mediated chromatin loop?

As described for the RNAPolII ChIP experiments above, we have been unable to gain reproducible results with the Rad21 antibody we have available to us.

12. By the authors own review (Ref 10), HPV episomal genome can be methylated. Is there any evidence that the E2 ORF CTCF binding site or other CTCF sites can be impacted by methylation. This is important as this could account for lack of CTCF binding during the HPV viral cycle. The authors should conduct COBRA or clonal bisulfite sequencing of the E2 ORF and other possible CTCF sites.

Our previous work demonstrated that CTCF binding to the HPV18 genome is not significantly altered during the HPV life cycle, while YY1 binding is reduced upon keratinocyte differentiation. In addition, the CTCF binding site in the HPV18 E2 ORF does not contain CpG dinucleotides and therefore CTCF binding will not be regulated by direct methylation. However, it has been shown that methylation of chromatin within several kilobases of CTCF binding sites in the human genome can affect binding [6]. It is therefore possible that methylation of the viral genome could result in reduced CTCF binding.

One interesting question that arises is whether CTCF binding to HPV episomes/integrants is reduced by increased DNA methylation of the HPV genome during carcinogenesis as it has been shown that CpG methylation in the E2, L2 and L1 regions of oncogenic HPV positively correlates with disease severity in the uterine cervix [7]. To begin to address this question, we have carried out pyrosequencing of bisulfite modified DNA to quantify CpG methylation of HPV18 in wild type and ΔCTCF-HPV18 HFKs from two independent donors. Results show a consistent increase in CpG methylation in the L2 and L1 regions of ΔCTCF-HPV18 episomes compared to wild type and we are currently working to determine the mechanism and biological significance of this. However, we believe that these experiments are outside the scope of the present study.

**Reviewer #3:** Fig. 1.
Lines 271-274 and Fig. 1A. Please show the details of the mutated sequences in the mapped CTCF binding site (nt 2960-3020) which is positioned at the N-terminal domain of E2. Hope the mutated sequences in the CTCF binding site are neutral (not being suppressive or enhancive to the viral genome expression!) and most importantly, will not inactivate the functional transactivation domain of E2, with normal E2 protein production.

We have now included a new figure (Fig.1A) detailing the position of the CTCF binding within HPV18 and the conservative (coding neutral) mutations that were introduced. These mutations were carefully designed to abrogate CTCF binding at the E2 ORF of viral episomes without altering the E2 protein coding sequence. We are therefore confident that E2 protein function cannot be altered by the mutations introduced in viral DNA. We have previously shown that these mutations do not alter E2 protein expression levels in organotypic raft cultures assessed by western blot analysis of raft lysates and immunofluorescence staining of raft sections ([5]; figure 9C and D therein). We have also previously demonstrated that the copy number of wild type and ΔCTCF-HPV18 episomes are equivocal in two independent keratinocyte donors by qPCR and Southern blotting [1, 5], indicating that the replication function of E2 is not altered. We now provide further data showing an equivocal level of differentiation-induced HPV18 episome amplification in both wild type and ΔCTCF-HPV18 keratinocytes in two independent donors (New Fig.2A and S2), and consistent E2 protein expression in raft cultures (new Fig.7G). Put together, these data confirm that E2 protein expression or function is not altered by the conservative mutations introduced in the E2 ORF.

Lines 280-285 and Fig. 1B. The increased splicing of 233^416 was most likely resulted from increased expression of delta CTCF genome because total viral reads were all increased across the entire virus genome! How did the authors confirm the same amount of recirculated wt and mt HPV18 DNA being transfected into the HFKs? Some quantitative verification data on input viral genome copies in transfected cells are needed at the beginning of the study.

While we agree that there is an overall increase in early transcript abundance when the E2-CTCF binding site is mutated, there is also an overall increase in 233^416 splicing in comparison to other splicing events. For example, splicing between 233^3434 is similar between wild type and mutant episomes (403 compared to 407), whereas splicing at 233^416 is almost doubled (16,557 compared to 28,918). We previously demonstrated an overall reduction in splicing from 233^3434 in ΔCTCF-HPV18 mutant episomes compared to wild type using conventional end-point RT-PCR [5]. Our RNA-Seq data suggest that this difference in splicing is due to a proportional increase in splicing at 233^416 compared to 233^3434.

We have now included Southern blot analysis of HPV18 wild type and ΔCTCF mutant in two independent donors showing equivocal episome copy number (New Fig 2A and S2).

Fig. 2C. How many repeats do you have? Is this just one sample data or one representative of three qualified Nanopore-seq?

The data shown in original Fig.2C (now Fig.2D) are taken from one high-depth Nanopore sequencing experiment. This figure is provided to demonstrate differential HPV18 promoter usage between undifferentiated and differentiated HFKs. The findings have been previously published and we include them here to validate our experimental model of keratinocyte differentiation.

We acknowledge the limitation in our sequencing data that are single sample reads. However, we make every effort to demonstrate that wild type HPV18 transcription is consistent with previously published reports. We also observed a high degree of concordance between Illumina and Nanopore data sets (compare Fig.1C to Fig.3). Furthermore, we validated the biological consequence of reduced differentiation-dependent late promoter activation in two independent donors (Fig.7). Nonetheless, we have now validated the reduction of 3165^3434 and 3284^3434 splicing in ΔCTCF-HPV18 episomes in comparison to WT by qRT-PCR in two independent donors (new Fig.4). These data confirm a significant reduction in 3165^3434 splicing in differentiated cells and a significant reduction in 3284^3434 splicing in both undifferentiated and differentiated cells.

Plus, all RNA-seq raw data should be submitted to NCBI Geo before manuscript submission.

The Illumina and Nanopore datasets described in this manuscript have now been submitted to the European Nucleotide Archive and an accession number included in our manuscript.

Fig. 3.
Lines 327-336 and Fig. 3 transcriptome summary. Additional methods should be applied to verify “novel transcripts” identified from this single sample study.

We have now increased the threshold for transcript identification in our Nanopore datasets to only include transcripts that were represented by at least three individual reads in at least two independent samples. This increased stringency is to ensure that each transcript species was identified multiple times in different sequencing runs and therefore unlikely to be a result of technical artefact. This greater stringency in our data reporting has removed many of the ‘novel’ transcripts we previously included. Although a total of four previously unreported transcripts were identified above this threshold and included in our updated Figure 3, these transcripts do not contain novel splice donor or acceptor sites, rather they contain a novel combination of promoter and splice site usage that can be fully characterised in our Nanopore data but not previously isolated using other methods.

Lines 337-339. Not sure about E2^E4L from the reported study (reference #38) but should be verified too in this report. Fig. 3. HPV18 transcripts of wt vs CTCF mt in HFKs under undifferentiated and differentiated conditions: As the reads are so low (many are less than 5 reads per million) in so called “new transcripts”, how did we know these splice-junction reads were not background noise reads. What were the bioinformatics perimeters and thresholds used in the analyses? Where are the verification data on these new transcripts? Obviously, the late transcripts are so low due to poor HFK differentiation under methylcellulose condition where the RNA was prepared for RNA-seq or Nanopore-seq.

Due to the increased stringency of our Nanopore data analysis, we have now removed transcripts encoding E2^E4L and putative E2^E4XL. However, the splicing identified in these transcripts (3165^3434 and 3284^3434, respectively) were also detected in E5 encoding transcripts #8 and #9 that originate from the P_3000_ promoter and therefore do not contain the E2 ATG. These transcripts were identified at ~8 rpm in HPV18 WT genome containing cells (both undifferentiated and differentiated samples) in our Nanopore data (Fig.3) and, importantly, the exact same splicing events were also identified in our Illumina based RNA sequencing (Fig.1C) and they have been previously reported in alternative HPV18 model systems and clinical samples [8, 9], giving confidence that they are biologically important. Interestingly, splicing at both 3165^3434 and 3284^3434 was appreciably less abundant in ΔCTCF-HPV18 genome containing cells compared to wild type in both Illumina and Nanopore datasets, giving further confidence in our overall conclusion that loss of CTCF binding in the E2 ORF affects downstream transcript splicing. These findings have now been further validated by qRT-PCR in two independent donors (New Fig.4).

Plus, all Nanopore-seq raw data should be submitted to NCBI Geo to obtain a Geo Accession number for manuscript inclusion and submission.

The Illumina and Nanopore datasets described in this manuscript have now been submitted to the European Nucleotide Archive and an accession number included in our manuscript.

Fig. 4 and Fig. 5. Because the RNA 5’ cap prevents linker ligation, RNA-seq and Nanopore-seq can’t be used to precisely map a transcription start site. What the authors’ reads counts in the two figures are the reads counts downstream of a real TSS.

We agree that Illumina and Nanopore RNA-Seq methods cannot be used to accurately map the transcriptional start site of each transcript. We acknowledged this in our original submission but to make it clear that we are broadly mapping promoter usage rather than specific TSSs, we have edited the manuscript throughout.

Fig. 4B. Since only one or two reads were identified at each nt position in the virus genome, it would be better to delete those reads or state clearly in text that the biological functions of these rare TSSs are minimal.

We have removed Fig.4B from the original manuscript as suggested.

Fig. 4D. If possible, please verify the promoter P3000 TSS by 5’ RACE.

The P_3000_ promoter has been previously identified in HPV18 using 5’ RACE [9]. Furthermore, a promoter at a similar position (nt3320/3335) has been identified by others in the related HPV31 [10, 11]. Our study is not an attempt to map novel promoters, but rather to assess the biological function of CTCF in regulating the activity of previously identified promoters. Our Nanopore data clearly show that transcripts that originate from this promoter are initiated within a broad nucleotide range (nt3401-4000), as has been previously reported [9], and that this promoter is activated upon keratinocyte differentiation, as has previously been reported [9].

Line 410-411 and Fig. 5. Need to be careful on the statement only until the authors ensure a functional E2 expression from ΔCTCF-HPV18 virus and the HFKs receiving the same amount of wt and mt HPV18 DNA.

We have now included data showing equivocal HPV episome copy number and E2 protein expression levels (New Fig.2A, S2 and 7G). The mutations we have introduced are deliberately conservative and therefore do not alter the protein sequence of the E2 protein. This is now better explained in the New Fig.1A.

What are reads-scales in the Fig. 5A and 5B? To this reviewer, both the P102 and the P811 promoters appear more active from the ΔCTCF-HPV18 virus than wt HPV18 virus in differentiated HFKs, despite that the authors claim only the P102, but not the P811, being more active in the differentiated HFKs. The difference in Fisher’s test must be showing more transactivation in ΔCTCF-HPV18 virus than wt HPV18.

The data in Fig.5 (now Fig.6) are represented in violin plots which show the probability density of data at a given value. We opted to show these data using violin plots in addition to the histogram representation of the same data in Fig.4 because the more commonly used box plots are not sufficient when assessing multimodal data (multiple peaks in distribution). Violin plots show the entire distribution of data including the presence of different peaks, their position and relative amplitude. Importantly, the amplitude (shape) of the kernel shows how tightly the data are distributed; wider sections of the violin plot represent a higher probability that members of the population will take on the given value, the skinnier sections represent a lower probability. In Fig.5 we can see that differentiation of the keratinocytes strongly activates the P_811_ promoter in HPV18 episomes; here the steep slope of the peak at P_811_ shows that the majority of the data are clustered here. In contrast, the shape of the kernel in the ΔCTCF-HPV18 dataset is broader and less steep, indicating less dense distribution and the difference between the early (P_102_) and late (P_811_) promoters is less pronounced. The depth of the kernels (y-axis) is not associated with transcript count, rather the maximum peak for each dataset. The data shown in Fig.4 (now Fig.5) shows the actual read counts at each 10nt position in the P_102_ and P_811_ promoters. The two datasets should be viewed together for a complete understanding of the differences.

Fig. 6 and Fig. 7.
Fig. 6. Please make the annotated genome positions in scale to relative H4Ac positions.

This figure has now been revised.

Where is your Fig. 6B (lines 24-426)?

We apologise for this oversight - reference for Fig.6B has now been removed.

Line 429. Not sure this statement is correct on reduction of P811 promoter activation from ΔCTCF-HPV18 virus in differentiated HFKs (Fig. 5B).

Please refer to our in-depth explanation of violin plots above.

Fig. 7D and 7E. Now we can see the sample variations when three biological samples were examined. Thus, the same principle should be applied to all RNA-seq and Nanopore-seq for the data shown in Figs. 1-5.

The data shown in Fig.7D (now Fig.7E) are from three independent raft cultures grown from two independent keratinocyte donors. While there is a lower amount of L1 positive cells in the rafts derived from keratinocyte donor 2 in comparison to 1, the effect of mutation of the E2-CTCF binding site is consistent; an almost complete loss of L1 protein expression is observed in both donors. Furthermore, we have now included data showing a consistent reduction in E1^E4 encoding transcript in ΔCTCF-HPV18 episomes following differentiation of two independent HFK donors (new Fig.7C).

The data shown in Fig.7E (now Fig.7F) corroborated the observed significant reduction of E1^E4 protein following methylcellulose differentiation of two independent keratinocyte donors in three independent raft cultures. We have now included E6 and E7 protein expression in these raft lysates along with E2 protein expression (Fig.7G).

We agree that it is best practise to carry out all experiments as independent repetitions where possible. However, the significant cost of doing this in all of our RNA-Sequencing experiments has prohibited us from doing so. Nonetheless, we have used two independent next generation sequencing methods (Illumina and Nanopore) which produced concordant results and we have included validation experiments (qRT-PCR, western blotting or immunofluorescence), which confirm that CTCF binding at the E2-ORF is necessary for transcript splicing at 3165^3434 and 3284^3434, and for robust late gene expression following differentiation in two independent HFK donors.

**Part III – Minor Issues: Editorial and Data Presentation Modifications**

**Reviewer #1:** (No Response)

**Reviewer #2:**

1. Line 134: splicing

Edited

2. Include antibody dilutions or amounts used in each application

Detailed information of antibodies used, and dilutions/amounts used in each application have now been included in the materials and methods section of the manuscript (lines 182 to 194).
3. Include amplicon size in Table 1

Details of amplicon size have now been included in Table 1. Upon reviewing the primer details originally listed in Table 1, we noticed that the reported mid-point was not correct; the nucleotide number was the 5’ annealing position of the forward primer in each pair. This has now been corrected to mid-point of the amplicon as we previously intended.

4. It is not obvious how images (Figure 7) were scored or analysed. Was it done by image analysis or by a researcher blinded to the experimental arm?

The slides were scored by 2 independent researchers blinded to the experimental arm; results were generally consistent between researchers and an average cell count was calculated.

5. A scale and 0- Z(?) x-co-ordinates should be provided on each HPV plot. At a suggestion, the CTCF site should be indicated with a black triangle or similar on Figure 1B & 6.

Corrected

6. Figure 1 ‘wild type’ is used, but HPV18 is used thereafter. Please be consistent with usage in all Figures and text.

Edited

7. Figure 1 Legend: ‘Next generation sequencing data were IGV.’ The sentence isn’t complete?

Edited

8. Figure 2B – number and label the exons. No mention of EMC1 isoform(?) numbers 1, 2 & 3 are mentioned in the legend.

Corrected

9. Lines 513-514 – ‘….in virus unable to bind CTCF’. Please rectify.

Corrected

**Reviewer #3:**

Introduction
Lines 97-103. In addition to CTCF, YY1 also interacts with HPV E7.

Reference to this study has now been included in out introduction (lines 115 to 119).

Line 105. It would be better to describe this promoter as P102 instead of P105 for HPV 18 E6/E7 and make it consistent in the entire manuscript. In fact, the main TSS in raft cultures starts from the P102, not much so from the P105.

This promoter is now described as P_102_ throughout the manuscript.

Lines 119-123. The full HPV18 transcriptome has been summarized in a JVI article (JVI 90: 9138-952, 2016) and host SRSF3 and hnRNPA1 regulation on HPV18 RNA splicing has been described in the same paper.

This article has now been cited in the revised article. We apologise for omitting discussion of this work in the original submission.

Discussion
Please make the promoter P102 as a name consistently in the entire manuscript, including figures and text.

This promoter is now defined as P_102_ throughout the manuscript.

Lines 469-483. These spliced transcripts are rare and should not be emphasized too much. In your best estimate, they are less than 10 reads per million both in undifferentiated and differentiated conditions (Fig. 3)!

As previously discussed, the threshold for inclusion of rare transcripts has been increased and discussion of E2^E4 encoding transcripts has been reduced accordingly.

Lines 484-509. Because the RNA-seq and Nanopore-seq lack qualified biological sample repeats and there was no data on functional E2 data from ΔCTCF-HPV18 virus, this paragraph is not relevant.

Although our RNA-Seq and Nanopore datasets do not include biological sample repetitions, they are in close agreement demonstrating that the differences in HPV transcript splicing and expression are consistent between two different biological samples and two independent methods. Furthermore, we have now included qRT-PCR validation on the reduction in SD3165 and SD3284 usage in ΔCTCF-HPV18 in two independent donors. We have also included data showing equal HPV genome replication (an important E2 function) and E2 expression levels. For these reasons, we have opted to leave the highlighted paragraph in our manuscript.

References:

[1]	Pentland I, Campos-Leon K, Cotic M, Davies KJ, Wood CD, Groves IJ, et al. Disruption of CTCF-YY1-dependent looping of the human papillomavirus genome activates differentiation-induced viral oncogene transcription. PLoS Biol 2018;16:e2005752.

[2]	Moody CA, Laimins LA. Human papillomaviruses activate the ATM DNA damage pathway for viral genome amplification upon differentiation. PLoS Pathog 2009;5:e1000605.

[3]	Smits P, Poumay Y, Karperien M, Tylzanowski P, Wauters J, Huylebroeck D, et al. Differentiation-dependent alternative splicing and expression of the extracellular matrix protein 1 gene in human keratinocytes. J Invest Dermatol 2000;114:718-724.

[4]	Zhang T, Cooper S, Brockdorff N. The interplay of histone modifications - writers that read. EMBO Rep 2015;16:1467-1481.

[5]	Paris C, Pentland I, Groves I, Roberts DC, Powis SJ, Coleman N, et al. CCCTC-binding factor recruitment to the early region of the human papillomavirus 18 genome regulates viral oncogene expression. J Virol 2015;89:4770-4785.

[6]	Wiehle L, Thorn GJ, Raddatz G, Clarkson CT, Rippe K, Lyko F, et al. DNA (de)methylation in embryonic stem cells controls CTCF-dependent chromatin boundaries. Genome Res 2019;29:750-761.

[7]	Wentzensen N, Sun C, Ghosh A, Kinney W, Mirabello L, Wacholder S, et al. Methylation of HPV18, HPV31, and HPV45 genomes and cervical intraepithelial neoplasia grade 3. J Natl Cancer Inst 2012;104:1738-1749.

[8]	Tan CL, Gunaratne J, Lai D, Carthagena L, Wang Q, Xue YZ, et al. HPV-18 E2^E4 chimera: 2 new spliced transcripts and proteins induced by keratinocyte differentiation. Virology 2012;429:47-56.

[9]	Toots M, Mannik A, Kivi G, Ustav M, Jr., Ustav E, Ustav M. The transcription map of human papillomavirus type 18 during genome replication in U2OS cells. PLoS One 2014;9:e116151.

[10]	Hummel M, Hudson JB, Laimins LA. Differentiation-induced and constitutive transcription of human papillomavirus type 31b in cell lines containing viral episomes. J Virol 1992;66:6070-6080.

[11]	Ozbun MA, Meyers C. Temporal usage of multiple promoters during the life cycle of human papillomavirus type 31b. J Virol 1998;72:2715-2722.
